# Machine learning-based predictive model for the differential diagnosis of ≤ 5 cm gastric stromal tumor and gastric schwannoma based on CT images

**DOI:** 10.1007/s12672-023-00801-4

**Published:** 2023-10-19

**Authors:** Guoxian Chen, Lifang Fan, Jie Liu, Shujian Wu

**Affiliations:** 1 School of Clinical Medicine, Wannan Medical College, Wuhu, China; 2 School of Medical Imageology, Wannan Medical College, Wuhu, China; 3 Department of Pediatric Surgery, Yijishan Hospital of Wannan Medical College, Wannan Medical College, Wuhu, 241000 China; 4 Department of Radiology, Yijishan Hospital of Wannan Medical College, Wannan Medical College, No.2 Zheshan West Road, Jinghu District, Wuhu, 241000 Anhui Province China

**Keywords:** Computed tomography, Machine learning, Gastric tumors, Gastric stromal tumor, Gastric schwannoma

## Abstract

The clinical symptoms of ≤ 5 cm gastric stromal tumor (GST) and gastric schwannoma (GS) are similar, but the treatment regimens are different. This study explored the value of computed tomography (CT) combined with machine learning (ML) algorithms to find the best model to discriminate them. A total of 126 patients with GST ≤ 5 cm and 35 patients with GS ≤ 5 during 2013–2022 were included. CT imaging features included qualitative data (tumor location, growth pattern, lobulation, surface ulcer status, necrosis, calcification, and surrounding lymph nodes) and quantitative data [long diameter (LD); short diameter (SD); LD/SD ratio; degree of enhancement (DE); heterogeneous degree (HD)]. Patients were randomly divided into a training set (n = 112) and test set (n = 49) using 7:3 stratified sampling. The univariate and multivariate logistic regression analysis were used to identify independent risk factors. Five ML algorithms were used to build prediction models: Support Vector Machine, k-Nearest Neighbor, Random Forest, Extra Trees, and Extreme Gradient Boosting Machine. The analysis identified that HDv, lobulation, and tumor growth site were independent risk factors (*P* < 0.05). We should focus on these three imaging features of tumors, which are relatively easy to obtain. The area under the curve for the SVM, KNN, RF, ET, and XGBoost prediction models were, respectively, 0.790, 0.895, 0.978, 0.988, and 0.946 for the training set, and were, respectively, 0.848, 0.892, 0.887, 0.912, and 0.867 for the test set. The CT combined with ML algorithms generated predictive models to improve the differential diagnosis of ≤ 5 cm GST and GS which has important clinical practical value. The Extra Trees algorithm resulted in the optimal model.

## Introduction

Gastric mesenchymal cell tumors account for about 3% of all gastric tumors, which mainly include the following four types: smooth muscle tumors (leiomyoma, glomus tumor, leiomyosarcoma), neurogenic tumors (schwannoma, neurofibroma, ganglioma, paraganglioma), fibroblast tumors (sclerofibroma, inflammatory myofibrocytoma), and gastrointestinal stromal tumors (GISTs) [1]. GISTs represent the prevailing gastric mesenchymal cell tumors, and despite their small size, they exhibit a substantial propensity for malignant transformation, with approximately 10% to 30% manifesting as malignant neoplasms [2–5]. Gastric stromal tumors (GSTs) constitute a significant proportion, ranging from 50 to 70%, of the overall GIST population [6], most of which are low-risk when the maximum diameter is less than 5 cm, but still have different levels of potential malignancy [7]. The predominant clinical manifestations of GSTs encompass abdominal discomfort and pain, accompanied by tumor hemorrhage and concomitant anemia. Less frequently observed symptoms include diminished appetite, weight loss, nausea, and obstruction of the esophagus. Prompt surgical excision upon diagnosis is typically the standard clinical approach for treating GSTs [8, 9]. Consequently, the timely identification, diagnosis, and management of GSTs play a pivotal role in determining patient prognosis. GSs account for 2–7% of gastric mesenchymal cell tumors [10], which are usually benign, rarely malignant, and mostly slow growing. When the tumors are small, they are usually asymptomatic. When symptoms are present, they mainly manifest as upper gastrointestinal bleeding caused by mucosal ulcers, atypical epigastric pain, or nonspecific dyspepsia, and direct endoscopic or laparoscopic radical resection can be effective treatment [11, 12]. In contrast, asymptomatic patients only need follow-up observation. The clinical symptoms of GST and GS ≤ 5 cm are similar with partial overlap in imaging findings [10, 13], but the treatment regimens are different, so the differential diagnosis of the two is particularly important to ensure proper treatment.

Machine learning (ML) is a relatively new category of artificial intelligence (AI), which has been widely used for many clinical applications [14–16]. In this study, we integrated advanced computed tomography (CT) imaging techniques possessing superior qualitative and quantitative attributes with five distinct machine learning (ML) algorithms. Our objective was to identify independent risk factors and develop an efficient prediction model capable of distinguishing between GST and GS with a diameter of ≤ 5 cm. The ultimate aim of this research is to offer surgeons a readily accessible and noninvasive tool for preoperative differential diagnosis. Notably, the accuracy of our prediction results surpasses that of subjective judgments made by radiologists.

## Materials and methods

### Study population

This study retrospectively analyzed patients treated at Yijishan Hospital of Wannan Medical College during 2013–2022 diagnosed with ≤ 5 cm GST or GS by surgery and pathology. Preoperative clinical data included sex, age, tumor markers [alpha fetoprotein (AFP), carcino-embryonic antigen (CEA), carbohydrate antigen 19-9 (CA19-9), and carbohydrate antigen 125 (CA125)]. The inclusion criteria were: (1) the available CT image was clear and could be used for all of the planned observations and measurements in the study; (2) the patient had not undergone radiotherapy, chemotherapy, or other non-surgical treatment before surgical resection; and (3) the patient had no multiple tumors excepting the primary gastric tumor and did not have concurrent gastric cancer. The exclusion criteria were: (1) incomplete imaging or clinicopathological data; (2) rupture or bleeding of the gastric tumor and (3) heavy respiratory artifacts or poor stomach filling. The patients were divided into a training set for modeling and internal verification and a test set for testing model stability and external verification. The method of stratified sampling according to 7:3 was adopted in the division of data sets to ensure that the two data sets have the same proportion of GST and GS to achieve data balance. All patients signed informed consent to participation in the study. This study was approved by the Ethics Committee of Wannan Medical College (IRB No. 199, 2023).

### Instruments and methods

Imaging was performed using Definition Flash dual-source CT or a Philips 64 spiral CT with tube voltage of 120 kV, tube current of 200 mA, slice thickness and spacing of 5 mm, and the pitch value was 0.6. Patients fasted for 6**–**10 h before the CT scan. Between 10 min before the scan, patients drank 800**–**1000 ml of warm water to fill the gastrointestinal tract. Throughout the scanning procedure, patients were in the supine position, and they were instructed to hold their breath before scans were initiated, which included a whole-abdomen plain CT scan and an enhanced three-phase scan. For the enhanced scan, first sweep imaging was performed. Next, a bolus of 80**–**100 ml iodixanol was delivered intravenously at a speed of 2.5 ml/s through the median cubital vein to allow dynamic contrast-enhanced scans, which were performed during the arterial phase, venous phase, and delayed phase at 30 s, 60 s, and 180 s, respectively, after the infusion was started.

### Image analysis

After scanning, the original images were sent to the Picture Archiving and Communication Systems (PACS). Two radiologists, with over 5 and 15 years of experience in abdominal imaging diagnosis, respectively observed images to assess the qualitative characteristics of tumors. In instances of disagreement, a senior physician was consulted to facilitate consensus. Qualitative analyses included tumor location, growth pattern, lobulation, surface ulcer status, necrosis, calcification, and surrounding lymph nodes. The following quantitative analyses were performed on the images at the maximum diameter of lesion shown in CT cross section: lengths of the long diameter (LD) and short diameter (SD), LD/SD ratio, and the degree of enhancement (DE), which was calculated as the difference between the CT value of each phase [arterial (DEa), venous (DEv), and delayed (DEd)] and the CT value of the plain scan. Tumor heterogeneous degree (HD) was measured and included as much of the tumor as possible without going beyond the tumor margin. The unevenness of plain scan phase, arterial phase, venous phase and delayed phase was represented by HDc, HDa, HDv, and HDd, respectively. HD was recorded as the standard deviation (SD) of the CT value in the region of interest (ROI). The quantitative indicators were assessed on three separate occasions by two physicians individually, and the mean values were subsequently considered as the ultimate outcomes.

### Feature screening and ML model construction and testing

The qualitative and quantitative features extracted from the enhanced CT images and clinical features of the tumors were analyzed by Least Absolute Shrinkage and Selection Operator (LASSO) regression to reduce the dimensionality. Prior to conducting LASSO regression analysis, the data underwent Z-Score standardization. This involved employing the specific standardization method denoted as z = (x−μ)/σ, where x represents the value of the random variable, μ denotes the population mean value, σ signifies the population standard deviation. Consequently, the standardized data exhibited a mean value of 0 and a variance of 1. Then, independent risk factors were further screened from potential risk factors using univariate and multivariate logistic regression analysis. Next, the independent risk factors were used to develop prediction models using the training set and five ML algorithms: Support Vector Machine (SVM), k-Nearest Neighbor (KNN), Random Forest (RF), Extra Trees (ET), and Extreme Gradient Boosting (XGBoost). All five models were evaluated in the test set to determine the optimal model.

### Statistical methods

Statistical analyses were performed with SPSS 23.0 and Python (3.5.6) software. The Shapiro–Wilk test was used to test the normality of the data, and data conforming to the normal distribution were used as $\overline{x}$ ± *s*, whereas non-normally distributed data were used as M50 (P25, P75) means; statistical comparisons were performed using Student’s *t* test or the Mann–Whitney *U* test, respectively. Categorical variables were analyzed using the *χ*^2^ test and Fisher’s exact test. LASSO regression analysis screened potential risk factors, and univariate or multivariate logistic regression analysis was used to screen independent risk factors. Odds ratio (OR) and 95% confidence interval (CI) were calculated for all independent risk factors. The diagnostic performance of the prediction models of the five ML algorithms was evaluated by the area under the curve (AUC), sensitivity analysis, specificity analysis, and accuracy of receiver operating characteristics (ROC) [17]. The AUC, sensitivity and specificity of the prediction model were determined by the Jorden index (Jorden index = sensitivity + specificity−1). The accuracy of the prediction model is the ratio of all the predicted accurate sample sizes to the total sample size [18]. Decision curve analysis (DCA) and confusion matrix were used to evaluate the clinical applicability and performance of the above models. A *P-*value < 0.05 was considered statistically significant.

## Results

### Characteristics of the training set and test set

In total, 126 patients with ≤ 5 cm diameter GST and 35 patients with GS were enrolled. Among the patients with ≤ 5 cm GST, there were 56 males and 70 females, aged 32–82 years (average 59.7 ± 9.9 years); 78 (62%) of the cases were very low or low risk (categorized as ‘low risk’ in this study), and 48 (38%) of the cases were medium or high risk (categorized as ‘high risk’ in this study). Among the patients with ≤ 5 cm GS, there were 8 males and 27 females, aged 33–76 years (average (58.4 ± 11.4 years), and all had benign tumors.

All of the quantitative and qualitative CT imaging characteristics and clinical features were compared between the training set and test set. Only the LD/SD ratio was statistically different between the two data sets, with a higher ratio of 1.17 in the training set compared with 1.14 in the test set (P = 0.04). All other characteristics were similar between the two groups (Table 1).

**Table 1:** Comparison of clinical imaging data between the training set and test set

Clinical image features	Training set (*n* = 112)	Test set (n = 49)	*t/*χ^2^/*Z* value	*P* value
Sex			2.505^a^	0.114
Male	40	24		
Female	72	25		
Age (years)	58.56 ± 10.15	61.37 ± 10.19	− 1.611^b^	0.109
AFP (ng/ml)	2.18 (1.66, 3.19)	2.24 (1.74, 3.04)	− 0.176^c^	0.860
CEA (ng/ml)	1.91 (1.28, 2.79)	1.65 (1.18, 2.86)	− 0.709^c^	0.478
CA199 (U/ml)	6.40 (3.13, 9.51)	7.62 (2.71, 11.20)	− 1.441^c^	0.150
CA125 (U/ml)	9.97 (7.93, 13.70)	10.30 (7.80, 14.45)	− 0.231^c^	0.817
Tumor site			5.364^a^	0.068
Cardia fundus	25	9		
Gastric body	66	37		
Gastric antrum	21	3		
Growth pattern			1.770^a^	0.413
Intracavity	68	28		
Mixed	24	8		
Extracavity	20	13		
Lobulation			3.447^a^	0.063
Yes	36	9		
No	76	41		
Superficial ulcer			1.146^a^	0.284
Yes	12	2		
No	100	47		
Necrosis			1.964^a^	0.161
Yes	40	12		
No	72	37		
Calcification			1.452^a^	0.228
Yes	6	6		
No	106	43		
Peripheral lymph node involvement			2.182^a^	0.140
Yes	16	3		
No	96	46		
LD (cm)	3.10 (2.10, 4.20)	2.50 (1.90, 3.60)	− 1.555^c^	0.120
SD (cm)	2.50 (1.80, 3.50)	2.20 (1.60, 3.10)	− 1.160^c^	0.246
LD/SD	1.17 (1.09, 1.29)	1.14 (1.06, 1.22)	− 2.054^c^	0.040
Dea (HU)	19.55 (13.18, 27.90)	18.10 (13.75, 26.65)	− 0.592^c^	0.554
Dev (HU)	35.30 (26.93, 43.90)	31.50 (25.40, 37.35)	− 1.336^c^	0.182
Ded (HU)	41.40 (33.20, 49.58)	35.60 (29.25, 43.60)	− 1.890^c^	0.059
HDc	8.30 (7.40, 9.43)	8.80 (6.85, 9.90)	− 0.709^c^	0.478
HDa	9.95 (8.40, 11.50)	10.70 (9.00, 12.25)	− 1.209^c^	0.227
HDv	10.60 (9.13, 13.10)	9.90 (9.00, 12.90)	− 0.467^c^	0.641
HDd	10.25 (8.90, 12.20)	10.50 (9.50, 12.15)	− 1.007^c^	0.314

^a^*χ*^2^ value

^b^*t* value

^c^*Z* value

### Feature screening

**Fig. 1:**
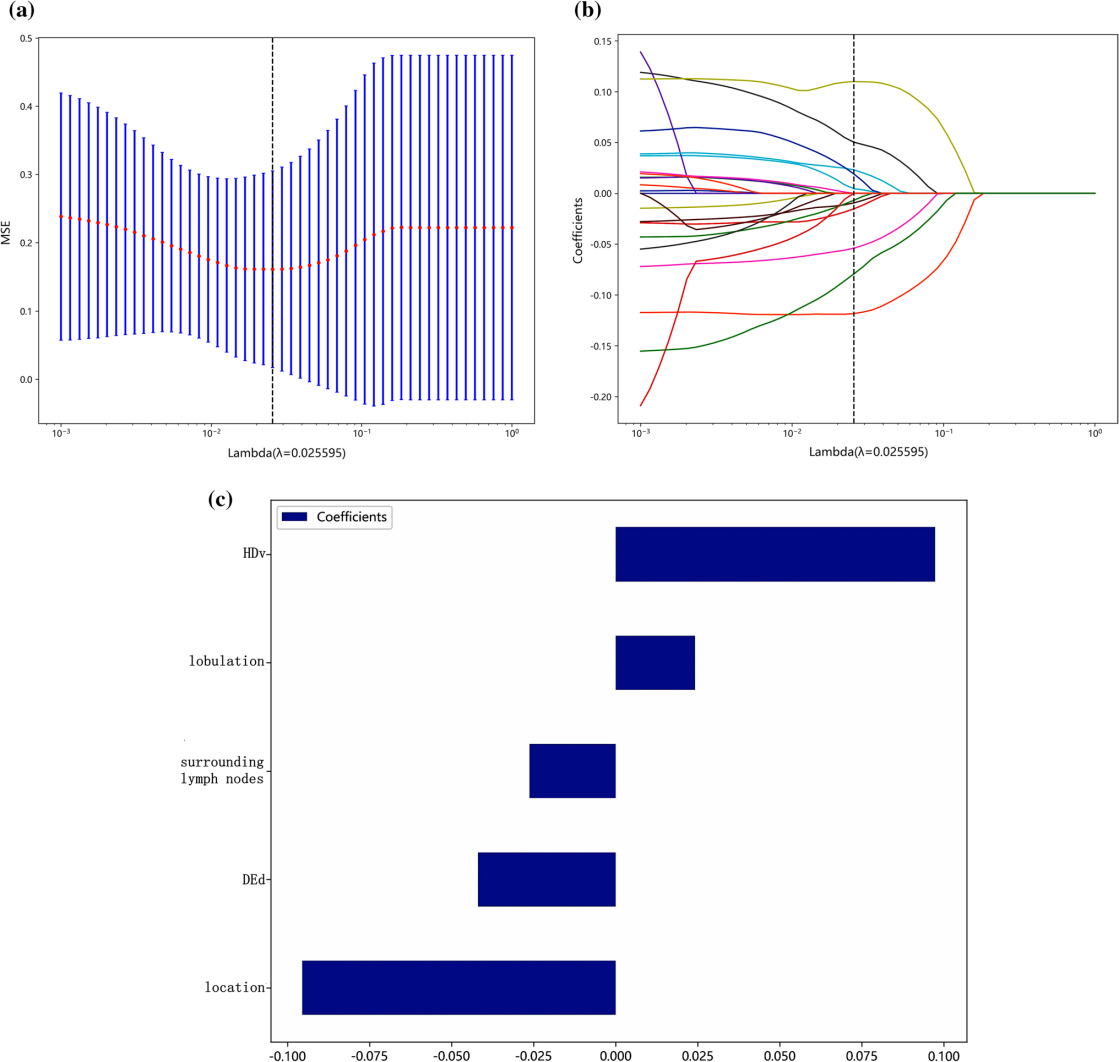
LASSO regression analysis identifies potential risk factors to discriminate tumor type. **a** LASSO regression analysis was performed with tenfold cross-validation to screen the clinical and imaging features for potential risk factors for GST ≤ 5 cm and GS ≤ 5 cm. **b** Distribution of regression coefficients for each clinical image feature. **c** Weights of the five potential risk factors that were screened out

**Fig. 2:**
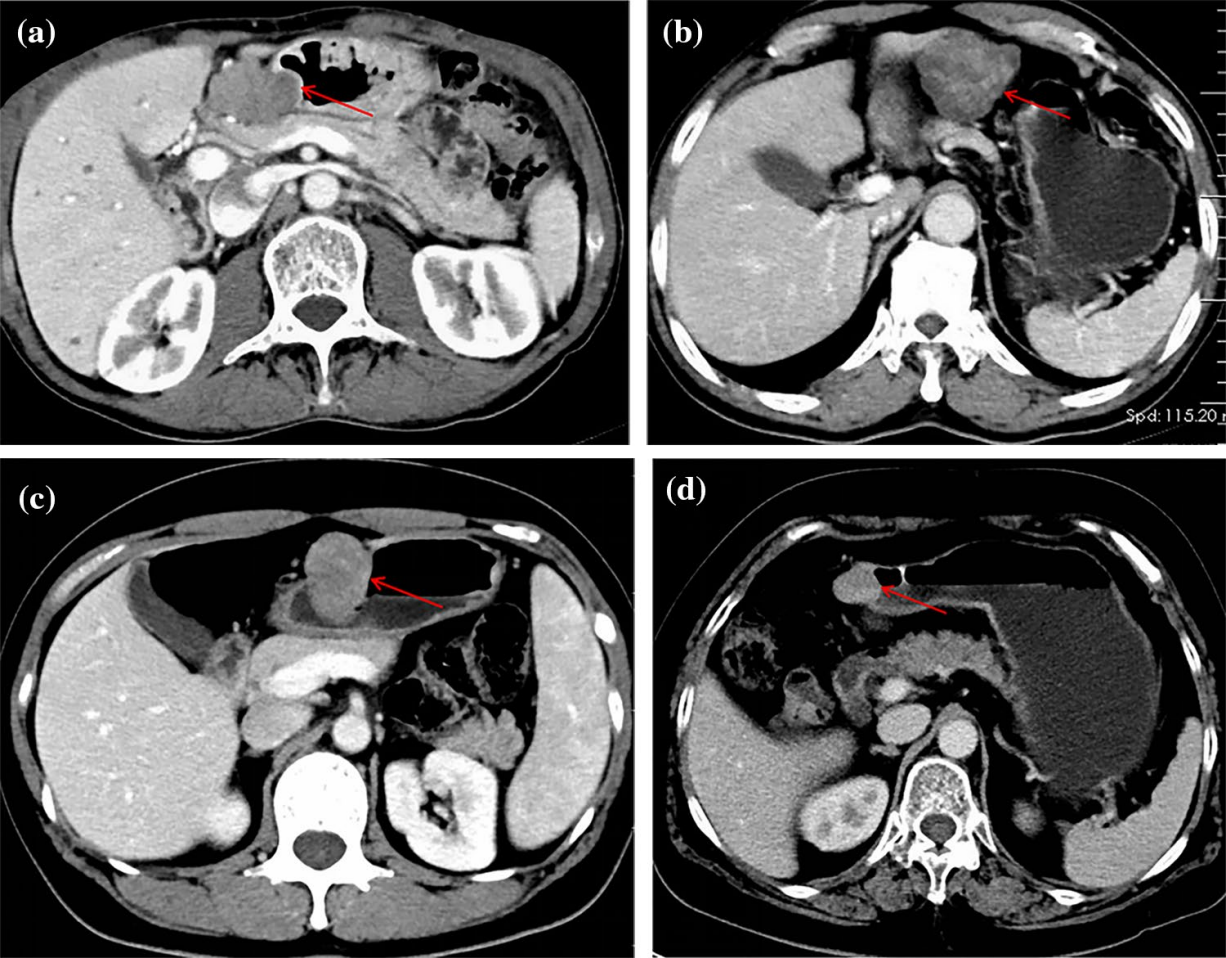
**a**, **b** were ≤ 5 cm GSTs. All the tumors showed growth outside the cavity, lobular changes and varying degrees of necrosis. **c**, **d** were GSs. All tumors are characterized by mixed growth, regular shape, uniform density, and round or oval mass

In the training set, LASSO regression was used for dimensionality reduction to screen the clinical and imaging features for potential risk factors for ≤ 5 cm GST and GS. This analysis identified that, among 23 clinical image features, when λ = 0.025595, tumor growth site, lobulation, peripheral lymph nodes, Hdv and DEd were potential risk factors; the other clinical image features were excluded (Figs. 1, 2). Using single-factor logistic regression, all five of the potential features were statistically significant (P < 0.05 for all comparisons), but multivariate logistic regression indicated that only HDv, lobulation, and tumor growth site were independent risk factors (P < 0.05) (Table 2).

**Table 2:** Univariate and multivariate logistic regression analysis of five potential risk factors screened by LASSO regression analysis

Potential risk factors	Univariate analysis	*P* value	Multivariate analysis	*P* value
	OR (95% CI)		OR (95% CI)	
HDv	1.736 (1.297, 2.325)	< 0.001	1.834 (1.238, 2.718)	0.002
Lobulation	5.077 (1.416, 18.206)	0.013	5.789 (1.092, 30.687)	0.039
Peripheral lymph node involvement	0.247 (0.082, 0.742)	0.013	0.244 (0.050, 1.196)	0.082
DEd	0.942 (0.905, 0.980)	0.003	0.947 (0.889, 1.008)	0.089
Growth site		0.001		0.005
Growth site (1)	0.155 (0.019, 1.246)	0.080	0.169 (0.017, 1.717)	0.133
Growth site (2)	0.031 (0.004, 0.276)	0.002	0.022 (0.002, 0.295)	0.004

### Model construction and testing

**Fig. 3:**
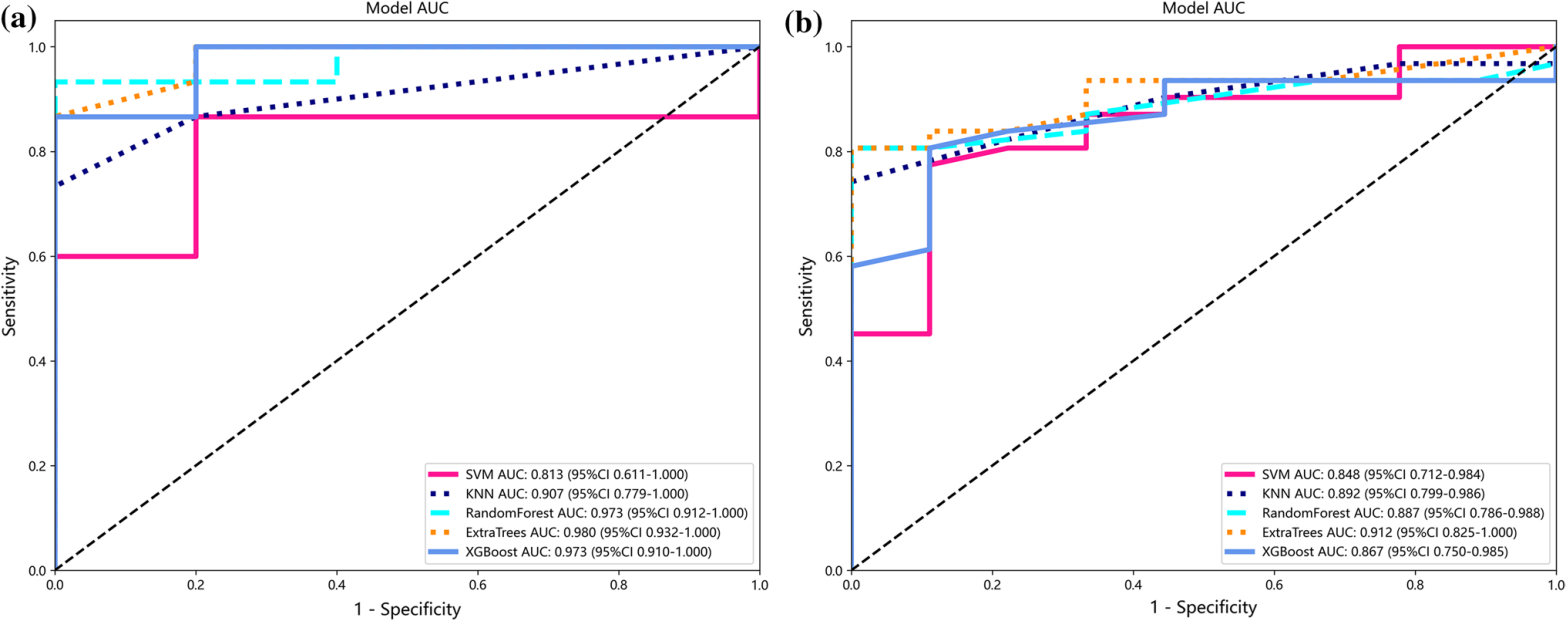
ROC curves of prediction models constructed by the five ML algorithms. **a** Performance of the models on the training set. **b** Performance of the models on the test set

**Fig. 4:**
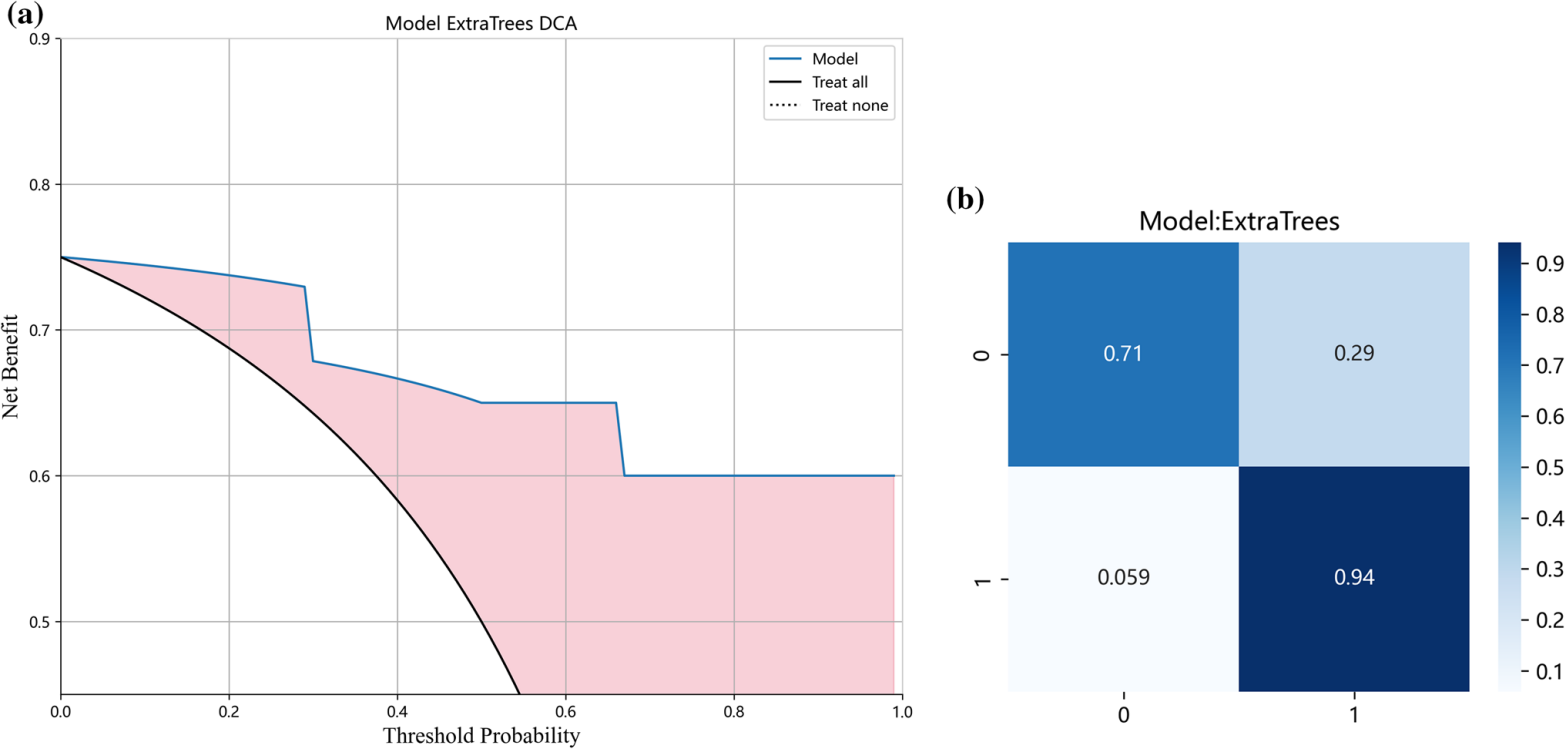
Clinical decision curve (**a**) and confusion matrix (**b**) of the test set when analyzed using the prediction model constructed by ET

To construct prediction models, these three independent risk factors were included in five ML algorithms and were evaluated. AUCs for the SVM, KNN, RF, ET, and XGBoost prediction models were, respectively, 0.790, 0.895, 0.978, 0.988, and 0.946 for the training set, and were, respectively, 0.848, 0.892, 0.887, 0.912, and 0.867 for the test set. Thus, the ET algorithm had the best performance (Table 3, Fig. 3). In this study, the sample size consisted of 126 GSTs and 35 GSs. The presence of an unbalanced sample size can lead to errors in prediction outcomes. The ET algorithm, known for its strong randomness, effectively mitigates the errors arising from data imbalance [19], resulting in smaller prediction errors. This could potentially explain the superior performance of the ET algorithm compared to other algorithms in this particular context. Using the prediction model constructed by ET algorithm as the output model, we then drew the DCA and confusion matrix of the test set. DCA showed the maximum net clinical benefit when the threshold probability was between 0 and 1, and the confusion matrix indicated that the model had good performance (Fig. 4).

**Table 3:** Evaluation of predictive models constructed by five ML algorithms

Category	AUC	95% CI	Accuracy	Sensitivity	Specificity
Training set					
SVM	0.790	0.689–0.890	0.785	0.811	0.692
KNN	0.895	0.839–0.952	0.868	0.611	1.000
RF	0.978	0.957–0.999	0.934	0.905	0.923
ET	0.988	0.976–1.000	0.950	0.874	1.000
XGBoost	0.946	0.902–0.989	0.901	0.905	0.846
Test set					
SVM	0.848	0.712–0.984	0.775	0.774	0.889
KNN	0.892	0.799–0.986	0.825	0.742	1.000
RF	0.887	0.786–0.988	0.825	0.806	1.000
ET	0.912	0.825–0.999	0.875	0.806	1.000
XGBoost	0.867	0.750–0.985	0.825	0.806	0.889

## Discussion

GST is the most common gastrointestinal stromal tumor (GIST), often occurring in middle-aged and elderly people, and with similar incidence rates in men and women [9]. The clinical symptoms are not specific, and, because only a small amount of submucosal tissue can be obtained by preoperative biopsy, it is difficult to accurately judge tumor heterogeneity [20]. In addition, preoperative biopsy is a common cause of tumor rupture and bleeding, leading to an increased risk of tumor dissemination. CT is an effective tool to support the diagnosis and differential diagnosis of gastrointestinal diseases [21]. GST and GS have similar clinical and imaging manifestations, whlie frequently difficult to discriminate. Therefore, non-surgical approaches to distinguish these two tumors are needed to ensure proper clinical management. In this study, a large number of clinical and CT features were inductively analyzed, the best features to identify the two tumor types were screened, and the three top-scoring features were input into five ML algorithms to construct prediction models to find the best model to distinguish small GST from GS.

Regardless of whether the dependent variable is continuous or categorical, LASSO regression can be applied by constructing a penalty function (λ) to eliminate low-correlation features and retain the optimal high-correlation features. In this study, five optimal features were screened out, including HDv, lobulation, peripheral lymph nodes, DEd, and tumor growth site.

HD is the standard deviation of CT in the tumor, and it is an indirect reflection of intratumoral heterogeneity. Tumors with different pathological bases have different degrees of heterogeneity ≤ 5 cm GSTs are low-grade malignant tumors or tumors with malignant potential, whereas GSs are almost always benign, and we found that these two GSMT subtypes are characterized by different HDs. However, only HDv qualified for inclusion in the prediction models, which may be owing to the fact that the amount of detectable heterogeneity between the two tumor types varies according to the post-enhancement phase, and that the heterogeneity between tumors is most prominent in the venous phase.

Malignant tumors grow faster than benign tumors, and the difference in proliferation rate of tumor cells results in irregular lobulated changes in the tumor body. Moreover, the higher the degree of malignancy, the greater the probability of lobulation [22]. GSs contain varying numbers of inflammatory cells, so reactive lymph nodes of different sizes often appear around the lesion, whereas ≤ 5 cm GSTs do not contain inflammatory cells, so this phenomenon is relatively rare. On enhanced scans, both tumors showed progressive enhancement, but the degree of progressive enhancement of ≤ 5 cm GSTs was lower than that of ≤ 5 cm GSs, and the peak was more anterior [23]. Thus the DE of the two tumor types after enhancement is different, and the difference becomes more obvious over time, which probably contributes to DEd being screened as an optimal feature. ≤ 5 cm GST and GS also have different predilection sites, with the former being likely to occur in the gastric body and gastric fundus [24], and the latter most commonly occurring in the gastric body followed by the gastric antrum and gastric fundus [25]. Univariate and multivariate logistic regression analysis of the five potential risk factors screened out using LASSO showed that HDv, lobulation, and tumor growth site were independent risk factors. This indicates that peripheral lymph nodes and DEd have some value in distinguishing small GSTs from GSs, but the value is limited.

The prediction models constructed by all five ML algorithms for the differential diagnosis of ≤ 5 cm GST and GS showed high efficiency. There have been other reports of using CT imaging data for the differential diagnosis of GISTs. Sun Jun [26] used the CT whole tumor histogram to identify 6 highly correlated histogram parameters; ROC curve was used to analyze the diagnostic efficiency of statistically significant parameters, and the highest AUC was 0.78. Wang Jian [27] used CT image features to identify ≤ 5 cm GST and GS, and found highly correlated features, ROC analysis resulted in a maximum AUC of only 0.674. The diagnostic efficiency and sample size of these previous studies were lower than this study. Our study incorporates new variables and uses a variety of ML algorithms to build an effective prediction model with improved performance on both the training set and the test set, which indicates the model is generalizable to other clinical samples. Wang [28] used CT images and ML to identify GSTs and GSs and found that the model constructed by logistic regression in the test set had the highest diagnostic efficiency, with an AUC of 0.967. Its diagnostic performance is better than that of ET algorithm in the test set (AUC = 0.912), and lower than that of ET algorithm in the training set (AUC = 0.988). However, it is not completely consistent with the conclusions of this study. In this study, a noninvasive differential diagnosis was performed to distinguish between the GST subgroup (≤ 5 cm) and GS. However, it is important to note that the ET algorithm has inherent limitations. Specifically, when the number of decision trees is substantial, the training time for the model becomes significantly prolonged. Consequently, in practical applications where real-time demands are paramount, the ET algorithm may not be the optimal choice.

This study was subject to several limitations. Firstly, the cases were exclusively obtained from a single hospital, resulting in an inadequate sample size. It is recommended to incorporate multicenter data for future investigations [29], which testing the generalization ability of the model, and gradually apply it in clinical practice [30]. Secondly, this study employed a retrospective analysis, which inherently introduces selection bias. Thirdly, all the included cases of GS were benign, thereby lacking the necessary imaging characteristics of rare malignant GSs. Consequently, the prediction models were unable to evaluate the features of malignant GSs. Lastly, the CT scans were conducted using the empirical method rather than the threshold method, potentially leading to periodic inconsistencies.

## Conclusion

The study showcased the utility of enhanced CT imaging features in distinguishing GSTs and GSs measuring ≤ 5 cm. The distinctive aspect of this research lies in the successful implementation of a predictive model, employing three optimal CT image features and the ET machine learning algorithm. Consequently, this approach offers surgeons a straightforward and non-intrusive means to develop an optimal treatment strategy for GIST patients prior to surgical intervention.

## Data Availability

The datasets generated during and/or analysed during the current study are available from the corresponding author (18895383277@163.com) on reasonable request.
